# Bevacizumab-Based Therapy Is Associated with Prolonged Progression-Free Survival in Patients with Peritoneal Mucinous Metastatic Colorectal Cancer

**DOI:** 10.3390/jcm15072805

**Published:** 2026-04-07

**Authors:** Süleyman Can, Veli Çakıcı, Gizem Bakır Kahveci, Şeyma Eroğlu, Burak Tok, Gökhan Uygun, Esra Özer, Yalçın Çırak, İvo Gökmen

**Affiliations:** 1 Division of Medical Oncology, Department of Internal Medicine, Faculty of Medicine, Çanakkale Onsekiz Mart University, Çanakkale 17100, Türkiye; suleycan@msn.com (S.C.); cakiciveli@gmail.com (V.Ç.); ozresra@hotmail.com (E.Ö.);; 2 Division of Medical Oncology, Department of Internal Medicine, Faculty of Medicine, Trakya University, Edirne 22030, Türkiye

**Keywords:** metastatic colorectal cancer, mucinous histology, bevacizumab, peritoneal metastasis, progression-free survival

## Abstract

**Objective:** In metastatic colorectal cancer (mCRC), mucinous histology has been associated with poor clinical outcomes, particularly in the presence of peritoneal metastasis. However, it remains unclear whether mucinous histology exerts a context-dependent effect on treatment outcomes by modifying the efficacy of anti-vascular endothelial growth factor (VEGF)-based therapies independently of metastatic dissemination patterns and chemotherapy backbone. **Methods:** We retrospectively analyzed 250 patients with mCRC treated with bevacizumab-containing systemic therapy. Tumors were classified as mucinous (*n* = 52) or non-mucinous (*n* = 198). Progression-free survival (PFS) and overall survival (OS) were estimated using the Kaplan–Meier method and compared using the log-rank test. Cox proportional hazards regression models were applied for univariate and multivariate analyses. Predefined subgroup analyses were conducted according to peritoneal metastasis status and chemotherapy backbone (oxaliplatin- or irinotecan-based). A 6-month landmark analysis was performed to reduce early progression bias. Interaction analyses evaluated potential effect modification between histology, peritoneal metastasis, and chemotherapy backbone. **Results:** Mucinous tumors were more frequently right-sided and strongly associated with peritoneal metastasis. In the overall cohort, mucinous histology was associated with significantly longer median PFS compared with non-mucinous histology (22.9 vs. 11.9 months; *p* < 0.001). This benefit was driven by patients with peritoneal metastasis, in whom mucinous histology was associated with markedly prolonged PFS (23.9 vs. 8.7 months; *p* < 0.001). No significant PFS difference according to histology was observed in patients without peritoneal metastasis. On multivariate analysis, mucinous histology remained independently associated with improved PFS (HR 0.44; 95% CI 0.25–0.78; *p* = 0.005), an effect preserved in the landmark cohort (HR 0.39; 95% CI 0.26–0.59; *p* < 0.001). A significant interaction between mucinous histology and peritoneal metastasis was observed (*p* for interaction = 0.040), indicating that the prognostic impact of histology differed according to metastatic pattern. No significant PFS difference or interaction was detected according to chemotherapy backbone within the mucinous subgroup. **Conclusions:** Among bevacizumab-treated patients with mCRC, mucinous histology—particularly in the presence of peritoneal metastasis—is associated with a pronounced PFS advantage independent of chemotherapy backbone. These findings suggest that mucinous peritoneal mCRC represents a biologically and clinically distinct subgroup that may derive context-specific and disproportionate benefit from anti-VEGF-based strategies, warranting prospective validation.

## 1. Introduction

Colorectal cancer (CRC) represents a growing global health burden, accounting for approximately 1.9 million new cases and more than 900,000 cancer-related deaths worldwide each year. Current projections estimate that this burden will increase substantially, reaching nearly 3.2 million new cases and 1.6 million deaths annually by 2040 [1]. The majority of CRC-related mortality arises from metastatic disease, highlighting the ongoing clinical need for effective, biologically informed treatment strategies capable of meaningfully prolonging survival in this setting. CRC is now widely recognized as a biologically heterogeneous disease characterized by distinct histological and molecular subtypes [2].

One of the most clinically notable manifestations of this heterogeneity is mucinous adenocarcinoma. Mucinous histology accounts for approximately 10–20% of all CRC cases and exhibits marked clinicopathological and biological differences compared with non-mucinous tumors [3]. This subtype is more frequently located in the right colon, occurs more commonly in female patients, and tends to be diagnosed at more advanced stages of disease [4]. Mucinous tumors are also strongly associated with higher rates of peritoneal carcinomatosis, largely attributable to their pronounced propensity for peritoneal dissemination compared with non-mucinous counterparts [5].

Although the prognostic significance of mucinous histology in early-stage disease remains controversial, studies conducted in the metastatic setting consistently indicate that this subtype is associated with a more aggressive clinical course and poorer survival outcomes [6,7]. In patients with metastatic mucinous CRC, lower response rates to cytotoxic chemotherapy and shorter progression-free survival (PFS) durations have been reported compared with non-mucinous cases [6]. These unfavorable outcomes are thought to be driven by the strong tendency of mucinous tumors toward peritoneal spread, the resulting limited opportunities for curative surgical intervention, and their relatively treatment-resistant biological characteristics [5].

At the molecular level, mucinous CRC is characterized by a higher prevalence of microsatellite instability-high (MSI-H) status, as well as increased frequencies of KRAS and BRAF mutations, alongside a lower incidence of TP53 mutations [5]. This distinct molecular profile is believed to contribute to the relatively chemoresistant biological phenotype observed in mucinous tumors when treated with conventional cytotoxic regimens. In contrast, data regarding the efficacy of targeted therapies in metastatic CRC with mucinous histology remain limited and inconsistent. While several studies suggest reduced effectiveness of anti-epidermal growth factor receptor (EGFR) therapies in this subgroup [8], the clinical impact of anti-angiogenic agents—particularly bevacizumab—within the context of mucinous tumor biology and peritoneal dissemination patterns has not been adequately elucidated. Moreover, the reliance of most existing studies on heterogeneous cohorts that do not stratify patients according to histological subtype or metastatic spread further complicates the clear assessment of treatment effects specific to mucinous histology [9].

Against this background, the present study aims to evaluate the impact of mucinous histology on progression-free survival in patients with metastatic colorectal cancer treated with bevacizumab, while explicitly accounting for the potential effect-modifying role of peritoneal metastasis through comprehensive subgroup and interaction analyses within a homogeneous real-world cohort.

## 2. Materials and Methods

### 2.1. Study Design

This study was designed as a dual-center, retrospective, observational cohort analysis based on real-world data, including patients with mCRC treated between January 2019 and December 2025 at the Faculty of Medicine Hospitals of Çanakkale Onsekiz Mart University and Trakya University. The primary endpoint of the study was PFS, and the secondary endpoint was OS.

### 2.2. Patient Population

Patients with histopathologically confirmed colorectal adenocarcinoma who were followed in the metastatic setting and received bevacizumab-containing systemic therapy at any line of treatment were included in the study. All eligible patients who received bevacizumab-based therapy during the study period were consecutively identified through institutional electronic medical record systems and included in the analysis. Tumors were classified as mucinous or non-mucinous according to histological assessments documented in pathology reports. Mucinous adenocarcinoma was defined in accordance with World Health Organization (WHO) criteria as tumors in which at least 50% of the lesion consisted of extracellular mucin. Histopathological classification was based on routine diagnostic pathology reports from each participating center, and no centralized pathological re-evaluation was performed. Histological classification was performed independently of treatment and survival outcomes.

The study cohort was defined by identifying patients who received bevacizumab-based therapy through electronic medical records; therefore, patients who did not receive bevacizumab were not captured within the study dataset.

### 2.3. Inclusion and Exclusion Criteria

The inclusion criteria were defined as:

(i) age ≥ 18 years,
(ii) presence of metastatic disease,
(iii) receipt of bevacizumab-based systemic therapy,
(iv) clearly documented histological subtype in pathology reports, and
(v) availability of follow-up data after treatment initiation.

Prior use of oxaliplatin- or irinotecan-based chemotherapy or other biological agents before bevacizumab treatment was not considered an exclusion criterion. Patients with incomplete clinical, pathological, or follow-up data, as well as those with a concurrent second primary malignancy, were excluded from the analysis.

### 2.4. Data Collection and Variables

Patient data were retrospectively retrieved from institutional electronic medical record systems. Collected variables included age, sex, body mass index, Eastern Cooperative Oncology Group (ECOG) performance status, de novo versus recurrent metastatic disease presentation, primary tumor location, and metastatic involvement patterns.

Tumor-related variables included histological subtype and available molecular characteristics, including RAS mutation status, BRAF V600E mutation status, and microsatellite instability (MSI) status. Treatment-related variables comprised the line of bevacizumab use and the accompanying chemotherapy backbone, classified as oxaliplatin-based or irinotecan-based regimens.

### 2.5. Treatment and Response Assessment

All patients received bevacizumab-containing systemic chemotherapy in accordance with routine clinical practice. Concomitant chemotherapy regimens were categorized as oxaliplatin-based or irinotecan-based. Treatment response was assessed using radiological imaging obtained during routine follow-up and evaluated according to Response Evaluation Criteria in Solid Tumors (RECIST), version 1.1. Responses were classified as complete response, partial response, stable disease, or progressive disease. Radiological assessments were generally performed at 8–12-week intervals.

Treatment regimens, dosing schedules, and number of cycles were determined according to routine clinical practice and physician discretion. Due to the retrospective nature of the study, detailed treatment exposure variables such as dose intensity and cumulative number of cycles were not uniformly available for analysis.

### 2.6. Endpoints

Progression-free survival was defined as the time from initiation of bevacizumab-based therapy to the date of radiological disease progression or death from any cause, whichever occurred first. OS was calculated as the time from treatment initiation to death from any cause or the date of last follow-up.

### 2.7. Ethics Approval

This study was approved by the Non-Interventional Clinical Research Ethics Committee of Çanakkale Onsekiz Mart University Rectorate (meeting date: 7 January 2026; meeting number: 2026-01/01-29; protocol number: 2026-01). The research protocol was reviewed and approved by the ethics committee, which determined that there were no ethical or scientific objections to conducting the study at the centers specified in the application file. The study was conducted retrospectively, and all data were analyzed in anonymized form without direct contact with participants. The research was carried out in accordance with the principles of the Declaration of Helsinki and Good Clinical Practice guidelines.

### 2.8. Statistical Analysis

Continuous variables were summarized as mean ± standard deviation or median (minimum–maximum), as appropriate, while categorical variables were reported as counts and percentages. Comparisons between groups were performed using Student’s *t*-test for continuous variables with approximately normal distributions and the Mann–Whitney U test for non-normally distributed variables. Categorical variables were compared using the chi-square test or Fisher’s exact test, as appropriate.

Survival outcomes were estimated using the Kaplan–Meier method, and differences between groups were compared using the log-rank test. The association between clinicopathological variables and survival outcomes was evaluated using the Cox proportional hazards regression model. Variables with a *p* value < 0.10 in univariable analyses, as well as clinically relevant covariates, were included in the multivariable models. The selection of variables was based on both statistical significance in univariable analyses and predefined clinical relevance to minimize potential confounding and avoid model overfitting. Results were reported as hazard ratios (HRs) with corresponding 95% confidence intervals (CIs). The proportional hazards assumption was assessed using Schoenfeld residuals. All covariates were analyzed as binary variables to enhance model stability and minimize the risk of overfitting.

To minimize potential time-dependent bias, a 6-month landmark analysis was performed for progression-free survival, including only patients who remained progression-free at the landmark time point. The prognostic impact of mucinous histology was re-evaluated within this cohort. Predefined subgroup analyses were conducted according to the presence of peritoneal metastases and the chemotherapy backbone (oxaliplatin- or irinotecan-based regimens). Potential interactions between mucinous histology, peritoneal metastasis, and chemotherapy backbone were assessed by including interaction terms in the Cox regression models, and *p* values for interaction were reported.

All statistical tests were two-sided, and a *p* value < 0.05 was considered statistically significant. Statistical analyses were performed using IBM SPSS Statistics software (version 26; IBM Corp., Armonk, NY, USA).

## 3. Results

### 3.1. Patient Demographic and Clinical Characteristics

A total of 250 patients with metastatic colorectal cancer who received bevacizumab-containing systemic therapy were included in the study. Of these, 52 patients (20.8%) had mucinous histology and 198 patients (79.2%) had non-mucinous histology (Table 1, which provides a detailed comparison of baseline clinicopathological characteristics between the two groups).

There were no statistically significant differences between the histological subgroups with respect to age, sex, BMI, or ECOG performance status. The proportion of patients aged ≥ 65 years was 50.0% in the mucinous group and 55.6% in the non-mucinous group (*p* = 0.474). Sex distribution was similar between groups (male sex: 67.3% vs. 61.1%; *p* = 0.412). No significant differences were observed across BMI categories (*p* = 0.491). An ECOG performance status ≥ 2 was comparable between the mucinous and non-mucinous groups (34.6% vs. 32.3%; *p* = 0.754). Primary tumor location differed significantly according to histological subtype. Mucinous tumors were more frequently located in the right colon compared with non-mucinous tumors (36.6% vs. 12.6%; *p* < 0.001), whereas left-sided colon and rectal tumors were more common in the non-mucinous group (Table 1).

Regarding metastatic dissemination patterns, peritoneal metastasis was significantly more frequent in the mucinous group (67.3% vs. 36.4%; *p* < 0.001). In contrast, liver (57.7% vs. 76.3%; *p* = 0.008) and lung metastases (42.3% vs. 62.6%; *p* = 0.008) were more common in the non-mucinous group. Ovarian metastases were observed more frequently in patients with mucinous histology (42.1% vs. 57.9%; *p* = 0.017). No significant differences were identified between groups with respect to bone (*p* = 0.944) or brain metastases (*p* = 0.915).

Molecular characteristics were also evaluated. Microsatellite instability–high status was detected in 37.5% of patients with mucinous histology and 62.5% of those with non-mucinous histology, with no statistically significant difference between groups (*p* = 0.177). The frequency of BRAF V600E mutation was similar between the two subgroups (27.3% vs. 72.7%; *p* = 0.435), as were RAS mutation rates (*p* = 0.873).

With respect to treatment characteristics, the use of bevacizumab as first-line or later-line therapy did not differ significantly between the histological subgroups (*p* = 0.869). The distribution of oxaliplatin- and irinotecan-based chemotherapy backbones was also comparable between groups (*p* = 0.202). In treatment response analyses, the rate of stable disease was numerically higher in the mucinous group (31.3% vs. 28.8%); however, no statistically significant differences were observed between groups in terms of objective response or progressive disease rates (overall response distribution *p* = 0.088) (Table 1).

### 3.2. Progression-Free Survival in the Overall Cohort

**Figure 1:**
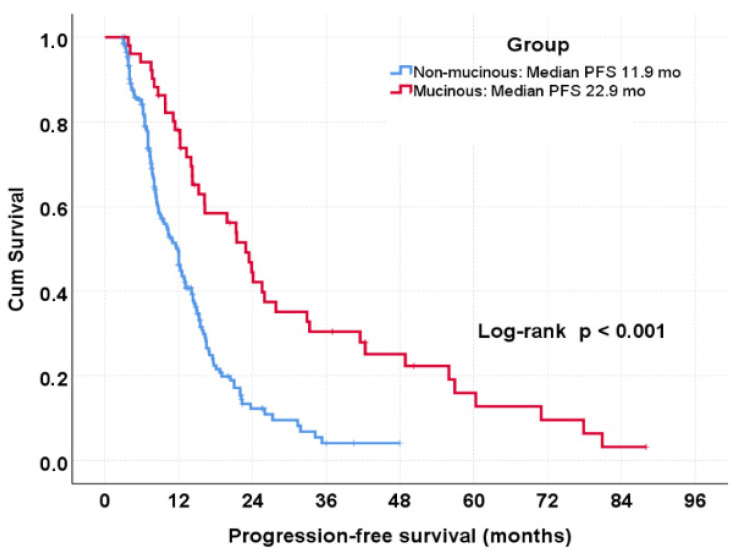
Kaplan–Meier curves for progression-free survival according to mucinous and non-mucinous histology in patients with metastatic colorectal cancer treated with bevacizumab-based therapy.

In the overall cohort, patients with mucinous histology experienced significantly longer progression-free survival compared with those with non-mucinous histology. Median PFS was 22.9 months in the mucinous group versus 11.9 months in the non-mucinous group (log-rank *p* < 0.001) (Figure 1, Table 2).

### 3.3. Cox Regression Analysis

In univariable Cox regression analysis, mucinous histology was significantly associated with prolonged progression-free survival (HR 0.39; *p* < 0.001). The presence of peritoneal metastasis (HR 0.66; *p* = 0.008) and de novo metastatic disease (HR 0.64; *p* = 0.005) were also associated with longer PFS, whereas liver metastasis was associated with an increased risk of progression (HR 2.35; *p* < 0.001). MSI-H status was similarly associated with improved PFS in univariable analysis (HR 0.35; *p* = 0.040). No significant associations with PFS were observed for age, sex, body mass index, ECOG performance status, primary tumor location, lung metastasis, RAS or BRAF mutation status, chemotherapy backbone, or line of bevacizumab use (Table 3).

In the multivariable Cox regression model including variables with *p* < 0.10 in univariable analysis, only mucinous histology remained independently associated with improved PFS (HR 0.44; *p* = 0.005). Peritoneal metastasis (*p* = 0.693), de novo metastatic disease (*p* = 0.264), and MSI-H status (*p* = 0.074) lost statistical significance, while liver metastasis showed borderline significance (HR 1.62; *p* = 0.079) (Table 3).

This finding was further supported by a 6-month landmark analysis performed to minimize early progression bias; in the landmark cohort, mucinous histology remained significantly associated with a reduced risk of progression (HR 0.39; 95% CI 0.26–0.59; *p* < 0.001).

### 3.4. Subgroup Analysis by Peritoneal Metastasis

Interaction analysis demonstrated a significant effect modification between mucinous histology and peritoneal metastasis on progression-free survival (p for interaction = 0.040), indicating that the prognostic impact of histology differed according to metastatic dissemination pattern (Table 4).

**Figure 2:**
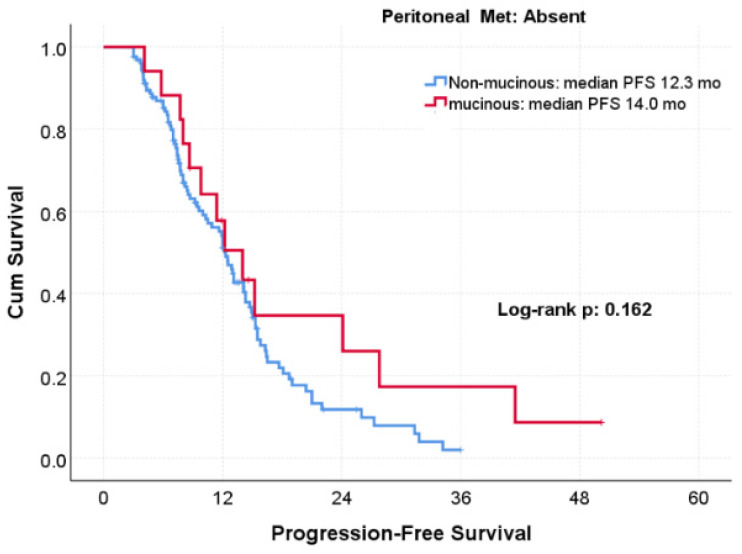
Progression-free survival according to mucinous versus non-mucinous histology in patients WITH peritoneal metastasis treated with bevacizumab-based therapy.

**Figure 3:**
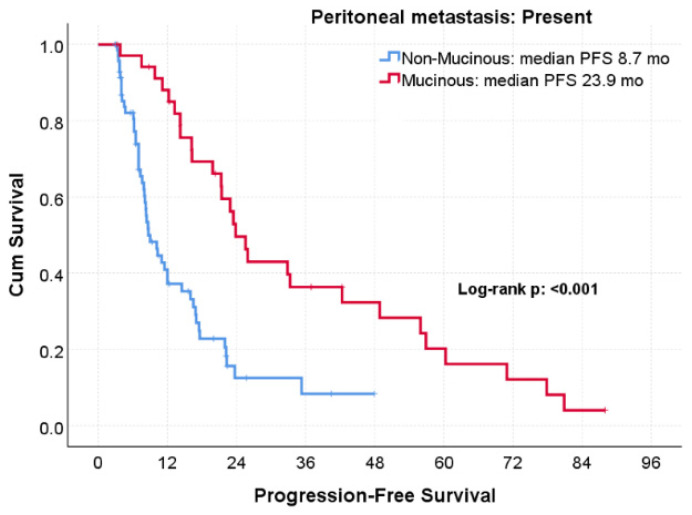
Progression-free survival according to mucinous versus non-mucinous histology in patients WITHOUT peritoneal metastasis treated with bevacizumab-based therapy.

The association between histology and PFS differed markedly according to peritoneal metastasis status. Among patients with peritoneal metastasis, mucinous histology was associated with a pronounced PFS benefit, with a median PFS of 23.9 months compared with 8.7 months in the non-mucinous group (log-rank *p* < 0.001; Figure 2). In contrast, no significant difference in PFS according to histology was observed among patients without peritoneal metastasis (14.5 vs. 12.3 months; *p* = 0.162; Figure 3) (Table 2).

### 3.5. Subgroup Analysis by Chemotherapy Backbone

When stratified by chemotherapy backbone, no significant difference in PFS was observed between oxaliplatin- and irinotecan-based regimens in the non-mucinous group (11.6 vs. 12.0 months; *p* = 0.863). In the mucinous subgroup, irinotecan-based regimens were associated with numerically longer PFS; however, this difference did not reach statistical significance (24.1 vs. 16.2 months; *p* = 0.190). Among patients with both mucinous histology and peritoneal metastasis, PFS was comparable regardless of chemotherapy backbone (irinotecan: 23.85 months; oxaliplatin: 23.45 months; *p* = 0.912) (Table 2).

### 3.6. Overall Survival: Cox Regression Analysis

In univariable Cox regression analysis, older age (≥65 years) was associated with an increased risk of death (HR 1.44; *p* = 0.022). De novo metastatic disease was strongly associated with improved overall survival (HR 0.43; *p* < 0.001), whereas liver metastasis was associated with increased mortality (HR 2.35; *p* < 0.001). Mucinous histology was associated with longer OS in univariable analysis (HR 0.65; *p* = 0.036). Lung metastasis showed borderline significance (HR 1.34; *p* = 0.071), and the use of bevacizumab in second or later lines showed a trend toward reduced mortality (HR 0.70; *p* = 0.066). No other clinical, molecular, or treatment-related variables were significantly associated with OS (Table 5).

In the multivariable Cox regression model including variables with *p* < 0.10 in univariable analysis, older age (HR 1.38; *p* = 0.041), de novo metastatic disease (HR 0.49; *p* < 0.001), liver metastasis (HR 1.80; *p* = 0.004), and lung metastasis (HR 1.41; *p* = 0.043) remained independently associated with OS. In contrast, mucinous histology lost its independent association with OS (*p* = 0.328). The use of bevacizumab in second or later lines remained of borderline significance in the multivariable model (HR 0.69; *p* = 0.062) (Table 5).

## 4. Discussion

In mCRC, mucinous adenocarcinoma has long been regarded as an unfavorable prognostic subtype. In patient cohorts treated with first-line oxaliplatin- and/or irinotecan-based chemotherapy, mucinous tumors have been shown to exhibit significantly lower objective response rates compared with non-mucinous counterparts (18% vs. 49%), along with markedly shorter median overall survival (~14 months vs. ~23 months) [10]. In multivariable analyses, mucinous histology has been consistently identified as an independent adverse prognostic factor, irrespective of performance status and metastatic burden [7]. From a clinicopathological and biological perspective, these tumors are more frequently right-sided and strongly associated with peritoneal metastasis [11], a distribution pattern that was likewise evident in our cohort, where mucinous tumors were predominantly right-sided and frequently accompanied by peritoneal dissemination (36.6% and 67.3%, respectively). In line with these characteristics, mucinous mCRC demonstrates reduced sensitivity to both cytotoxic chemotherapy and targeted agents; notably, even in RAS/BRAF wild-type disease, responses to anti–EGFR therapies remain limited, with substantially lower objective response rates (4% vs. 51%) and shorter median progression-free survival (2.8 vs. 6.7 months) [12].

Despite this unfavorable biological framework, our cohort provides evidence that the prognostic relevance of mucinous histology may be modified by the systemic treatment context. Among patients with metastatic disease treated with bevacizumab-containing regimens, the progression-free survival (PFS) difference between mucinous and non-mucinous subgroups was markedly attenuated compared with historical series. Notably, in this real-world bevacizumab-treated population, median PFS reached 22.9 months in patients with mucinous histology, whereas it remained limited to 11.9 months in the non-mucinous group, suggesting that anti–VEGF–based strategies may partially counterbalance the adverse biological features traditionally attributed to mucinous colorectal cancer.

Our findings are consistent with a large multicenter analysis including 685 patients with mCRC treated with first-line bevacizumab plus chemotherapy, in which no significant difference in median overall survival was observed between mucinous and non-mucinous cases (28.2 months vs. 27.7 months; HR = 0.92; *p* = 0.53) [6]. Although objective response rates were lower in the mucinous subgroup (41.5% vs. 62.4%), the comparable survival outcomes suggest that anti–VEGF therapy may partially offset the inherent biological disadvantage of mucinous tumors. This observation is further supported by analyses reporting pronounced PFS benefit with bevacizumab-based regimens in the CMS4 molecular subtype—characterized by dominant stromal/mesenchymal features and frequent mucinous histology—where irinotecan-based chemotherapy was associated with a substantial reduction in the risk of progression (PFS HR ~0.31) [13].

Peritoneal carcinomatosis represents one of the poorest prognostic clinical subgroups in mCRC. In the presence of peritoneal metastasis, overall survival is significantly shorter compared with liver-limited metastatic disease (median OS ~16.3 months vs. 19.1 months), and the risk of death increases by approximately 1.4-fold in cases with multiorgan involvement [14]. In this patient population, the likelihood of achieving curative systemic therapy or conversion surgery is low [15]. Peritoneal metastasis is also associated with aggressive molecular features; notably, the BRAF V600E mutation is more frequently observed in this subgroup and is widely regarded as a strong negative prognostic biomarker [16]. A strong association between mucinous histology and peritoneal dissemination has likewise been demonstrated in large patient series [16]. Consistent with these observations, peritoneal involvement in our cohort tended to occur within a distinct metastatic pattern characterized by less frequent hematogenous spread to the liver and lungs, suggesting that spatial dissemination patterns may reflect underlying biological differences rather than disease burden alone.

In our study, the clinical impact of bevacizumab therapy became most evident in the biologically and clinically most disadvantaged subgroup, defined by the coexistence of mucinous histology and peritoneal metastasis. In the presence of peritoneal metastasis, patients with mucinous tumors treated with bevacizumab-based therapy achieved a median PFS of 23.9 months, whereas PFS remained limited to 8.7 months in non-mucinous cases. Notably, this difference emerged predominantly during the early phase of treatment; in the 6-month landmark analysis, the risk of progression was significantly lower in the mucinous subgroup (HR 0.39; *p* < 0.001), suggesting improved early disease control with anti–VEGF therapy in this biological context.

Direct comparisons with anti–epidermal growth factor receptor (EGFR)–based therapies were not feasible in our cohort, as all patients received bevacizumab-based treatment. Nevertheless, our findings are consistent with prior evidence from a large registry-based analysis, in which first-line bevacizumab-containing regimens were associated with longer PFS (median ~9.6 vs. 6.1 months) and overall survival (OS) (26.3 vs. 12.7 months) compared with anti-EGFR–based therapies among patients with metastatic colorectal cancer and peritoneal metastasis, whereas no such difference was observed in patients without peritoneal involvement [17].

Within this context, the unexpectedly prolonged PFS observed in mucinous tumors with peritoneal metastasis in our cohort provides further support for a potentially clinically meaningful benefit of anti-VEGF therapy in this distinct biological subgroup.

Peritoneal carcinomatosis develops within a largely VEGF-dependent tumor microenvironment characterized by intense angiogenesis, increased vascular permeability, and pronounced hypoxia [18]. Tumor cells implanted on peritoneal surfaces promote the secretion of VEGF and other pro-angiogenic factors, thereby driving both neovascularization and vascular leakiness; this process plays a central role in the progression of peritoneal metastasis and the development of malignant ascites [19]. These biological features provide a strong rationale for a potential therapeutic advantage of anti-vascular endothelial growth factor (VEGF) therapies in patients with mCRC exhibiting peritoneal dissemination.

VEGF inhibition mediated by bevacizumab may exert its effects in peritoneal metastatic disease through two principal mechanisms: (i) suppression of angiogenesis, leading to delayed growth of tumor nodules, and (ii) reduction in vascular permeability and interstitial pressure, thereby enhancing the penetration of chemotherapeutic agents into tumor tissue. Experimental models have demonstrated that inhibition of VEGF signaling within the peritoneal carcinomatosis milieu reduces intratumoral pressure, improves the distribution of cytotoxic agents, and limits tumor growth [19].

Mucinous adenocarcinomas are characterized by abundant extracellular mucin accumulation, relative hypovascularity, and marked hypoxia—features that have been associated with resistance to cytotoxic chemotherapy [7]. Attenuation of hypoxia-driven VEGF activation and partial “normalization” of aberrant tumor vasculature may therefore constitute a biological framework through which bevacizumab enhances the efficacy of chemotherapy in tumors with mucinous histology and peritoneal spread [20]. The pronounced PFS benefit observed in mucinous patients with peritoneal metastasis in our study appears biologically concordant with these microenvironmental mechanisms.

Our subgroup analyses demonstrated that the PFS benefit observed in patients with mucinous histology was independent of the chemotherapy backbone administered. Among mucinous patients treated with bevacizumab in combination with either irinotecan-based or oxaliplatin-based regimens, no statistically significant difference in PFS was observed (24.1 vs. 16.2 months; *p* = 0.190). This finding was even more pronounced in the mucinous + peritoneal metastasis subgroup, in which similarly prolonged PFS durations were achieved regardless of the chemotherapy backbone (23.85 months vs. 23.45 months; *p* = 0.912). These results suggest that the observed clinical benefit is attributable primarily to the shared contribution of bevacizumab rather than to the superiority of a specific cytotoxic agent. In the pre-bevacizumab era, irinotecan-based regimens were reported to confer more favorable outcomes in mucinous tumors [10]; however, this advantage appears to have diminished in the anti-VEGF era. Indeed, in a large multicenter analysis of bevacizumab-treated patients, no significant survival difference was observed between FOLFIRI and FOLFOX in mucinous cases, with median overall survival of approximately 28 months reported in both arms [6]. Collectively, our real-world cohort corroborates that the favorable impact of mucinous histology on PFS is independent of chemotherapy type.

Importantly, interpretation of the multivariable survival analyses further supports the robustness of these findings. In the multivariate model for progression-free survival, mucinous histology emerged as the only variable independently associated with a reduced risk of progression, while factors such as peritoneal metastasis, de novo disease presentation, and MSI-H status lost statistical significance after adjustment. This suggests that the observed PFS benefit is not simply a surrogate of metastatic burden or molecular enrichment, but rather reflects a distinct biological behavior associated with mucinous histology in the context of bevacizumab-based therapy.

Although molecular characteristics such as RAS, BRAF V600E, and MSI status are known to influence prognosis in metastatic colorectal cancer, these variables were not independently associated with progression-free survival in our multivariable analysis. This suggests that the observed benefit associated with mucinous histology is unlikely to be solely driven by underlying molecular differences.

In contrast, overall survival was predominantly driven by established clinical determinants, including age and hematogenous metastatic spread to the liver and lungs, whereas mucinous histology did not retain independent prognostic significance in the multivariable OS model. This divergence between PFS and OS indicates that the benefit associated with mucinous histology primarily manifests as improved disease control rather than prolonged survival, underscoring the importance of distinguishing progression dynamics from ultimate survival outcomes. This discrepancy between progression-free survival and overall survival may also reflect the influence of post-progression treatments and subsequent lines of therapy, which were not captured in this retrospective analysis.

The mucinous histology × peritoneal metastasis interaction identified in our study provides a clinically meaningful insight. Interaction analysis demonstrated that, when considered jointly, these two variables were associated with a significant reduction in the risk of progression (HR 0.45; 95% CI 0.21–0.97; *p* = 0.040). Notably, in the absence of peritoneal metastasis, no significant difference in progression-free survival (PFS) was observed between mucinous and non-mucinous histology (14.0 vs. 12.3 months; *p* = 0.162), whereas in the presence of peritoneal metastasis, mucinous histology conferred a pronounced PFS advantage (23.9 vs. 8.7 months; *p* < 0.001). Furthermore, the significantly lower risk of progression observed in the mucinous subgroup in the 6-month landmark analysis (HR 0.39; *p* < 0.001) suggests that this benefit emerges early during treatment and reflects sustained disease control. Collectively, these findings indicate that the prognostic impact of mucinous histology is context-dependent and support the notion that bevacizumab-based therapies may represent a rational therapeutic strategy in patients with mCRC and peritoneal metastasis.

In contrast, the absence of a significant PFS advantage in mucinous tumors without peritoneal metastasis may be explained by differences in metastatic microenvironment and VEGF dependency. Peritoneal dissemination is characterized by a highly angiogenic and VEGF-driven milieu, whereas hematogenous metastatic sites such as the liver and lungs are less dependent on VEGF-mediated vascular permeability and angiogenesis.

Additionally, mucinous tumors exhibit abundant extracellular mucin, relative hypovascularity, and impaired drug penetration, which may limit the effectiveness of systemic therapies in non-peritoneal settings. In the absence of a VEGF-dominant microenvironment, the potential benefits of bevacizumab, including vascular normalization and enhanced drug delivery, may be attenuated.

These findings support the hypothesis that the therapeutic impact of anti-VEGF strategies in mucinous colorectal cancer is strongly context-dependent and largely restricted to peritoneal metastatic disease.

Patients with mucinous histology mCRC represent an underrepresented population in the literature, in whom detailed subgroup analyses have been limited in most prior studies. The majority of available data have focused on the overall prognostic impact of mucinous histology, whereas studies evaluating clinical outcomes in the anti-VEGF era—particularly within the peritoneal metastasis subgroup—remain scarce. In this context, our study addresses an important gap in the literature by systematically examining the clinical impact of mucinous histology within a homogeneous cohort in which all patients received bevacizumab-based therapy. Our findings diverge substantially from the analysis reported by Mekenkamp et al., who identified mucinous histology as an independent adverse prognostic factor for overall survival (HR ~1.7); however, in that study, patients were treated across heterogeneous therapeutic regimens, precluding assessment of the potential mitigating effect of anti-VEGF therapy [2]. In contrast, within the present cohort uniformly treated with bevacizumab, mucinous histology did not confer a progression-free survival disadvantage in the overall population, and notably, in the presence of peritoneal metastasis, median PFS was significantly longer in mucinous cases compared with non-mucinous tumors. This observation is consistent with findings from large multicenter analyses in which no overall survival difference was observed between mucinous and non-mucinous subgroups among bevacizumab-treated patients [7].

Conversely, studies reporting a poor response of the mucinous subtype to anti–epidermal growth factor receptor (EGFR) monoclonal antibodies [12] suggest that bevacizumab-based strategies may represent a rational first-line therapeutic choice in this patient population. Indeed, real-world data demonstrating the superiority of bevacizumab-containing combinations over cetuximab-based regimens in patients with peritoneal metastasis—with longer progression-free survival (9.6 vs. 6.1 months) and overall survival (26.3 vs. 12.7 months)—lend further support to this approach [21]. The frequent association of mucinous tumors with the CMS4 molecular subtype, characterized by hypoxia, stromal activation, and enhanced angiogenesis, also provides a strong biological rationale for anti-VEGF strategies in this setting [22]. Within this context, our study offers hypothesis-generating real-world evidence to inform treatment optimization in patients with mucinous mCRC, particularly those with peritoneal metastasis.

### Strengths and Limitations

One of the principal strengths of this study is the inclusion of a relatively homogeneous patient population uniformly treated with bevacizumab-based therapy. The use of similar chemotherapy backbones in combination with bevacizumab across the cohort substantially reduced treatment-related confounding effects. The proportion of patients with mucinous histology was consistent with existing literature, and the adequate representation of patients with peritoneal metastasis enabled clinically meaningful and well-powered subgroup analyses. The comprehensive evaluation of progression-free survival according to histology, metastatic dissemination patterns, and chemotherapy backbone, together with the incorporation of interaction analyses, further strengthens the methodological rigor of the study.

Nevertheless, several limitations should be acknowledged. The retrospective, dual-center design inherently increases the risk of selection bias and limits the generalizability of the findings. Histological classification was based on institutional pathology reports, and no centralized pathological re-review was performed. Incomplete availability of molecular data across the cohort limited the ability to fully adjust for potential molecular confounders. The relatively small number of patients in the mucinous subgroup, particularly after stratification by peritoneal metastasis, may have limited statistical power. In addition, the assessment of radiological progression in patients with peritoneal metastasis is inherently challenging and may introduce a degree of uncertainty in progression-free survival estimation. Data on post-progression treatments and potential crossover effects were not available and therefore could not be accounted for in the analyses. Accordingly, the present findings should be considered hypothesis-generating and warrant confirmation in prospective, multicenter studies.

Taken together, these limitations highlight the need for prospective, multicenter studies to validate and further refine these findings.

## 5. Conclusions

This retrospective analysis demonstrates that the impact of mucinous histology on PFS in patients with mCRC treated with bevacizumab-based therapy is strongly dependent on the pattern of metastatic dissemination. In particular, the observation of a pronounced PFS advantage among patients with mucinous tumors in the presence of peritoneal metastasis suggests that this subgroup may derive a disproportionate clinical benefit from anti-angiogenic strategies.

Importantly, the preservation of this benefit irrespective of the accompanying chemotherapy backbone supports the notion that the observed clinical advantage is primarily driven by the contribution of bevacizumab rather than by differences in cytotoxic regimens. These findings highlight mucinous peritoneal mCRC as a biologically and clinically distinct disease subset and underscore the relevance of integrating histological subtype with metastatic pattern in therapeutic decision-making.

If validated in prospective studies, this context-dependent effect may enable further refinement of personalized treatment strategies for patients with mucinous metastatic colorectal cancer, particularly those presenting with peritoneal involvement.

## Figures and Tables

**Table 1:** Baseline demographic, clinicopathological, molecular, and treatment characteristics of metastatic colorectal cancer patients treated with bevacizumab according to histology.

Variable	Total Cohort (*n* = 250)	Mucinous (*n* = 52)	Non-Mucinous (*n* = 198)	*p* Value
**Demographic characteristics**				
Age < 65 years, *n*	114	26 (22.8%)	88 (77.2%)	0.474
Age ≥ 65 years, *n*	136	26 (19.1%)	110 (80.9%)	
Male sex, *n*	156	35 (22.4%)	121 (77.6%)	0.412
Female sex, *n*	94	17 (18.1%)	77 (81.9%)	
BMI < 25 kg/m^2^, *n*	98	17 (17.3%)	81 (82.7%)	0.491
BMI 25–29.9 kg/m^2^, *n*	99	24 (24.2%)	75 (75.8%)	
BMI ≥ 30 kg/m^2^, *n*	53	11 (20.8%)	42 (79.2%)	
**Clinical status**				
ECOG 0–1, *n*	168	34 (20.2%)	134 (79.8%)	0.754
ECOG ≥ 2, *n*	82	18 (22.0%)	64 (78.0%)	
**Primary tumor characteristics**				
Right colon, *n*	71	26 (36.6%)	45 (63.4%)	<0.001
Left colon, *n*	87	11 (12.6%)	76 (87.4%)	
Rectum, *n*	92	15 (16.3%)	77 (83.7%)	
**Molecular characteristics**				
MSI-H †, *n*	8	3 (37.5%)	5 (62.5%)	0.177
MSS/MSI-L †, *n*	143	26 (18.2%)	117 (81.8%)	
RAS mutation present, *n*	164	34 (20.7%)	130 (79.3%)	0.873
BRAF V600E mutation present †, *n*	11	3 (27.3%)	8 (72.7%)	0.435
**Disease presentation and metastatic pattern**				
De novo metastatic disease, *n*	153	30 (19.6%)	123 (80.4%)	0.560
Recurrent metastatic disease, *n*	97	22 (22.7%)	75 (77.3%)	
Peritoneal metastasis present, *n*	107	35 (32.7%)	72 (67.3%)	<0.001
Liver metastasis present, *n*	181	30 (16.6%)	151 (83.4%)	0.008
Lung metastasis present, *n*	146	22 (15.1%)	124 (84.9%)	0.008
Ovarian metastasis present, *n*	19	8 (42.1%)	11 (57.9%)	0.017
Bone metastasis present, *n*	52	11 (21.2%)	41 (78.8%)	0.944
Brain metastasis present, *n*	9	2 (22.2%)	7 (77.8%)	0.915
**Treatment characteristics**				
Bevacizumab 1st-line, *n*	198	41 (20.7%)	157 (79.3%)	0.869
Bevacizumab ≥ 2nd-line, *n*	51	11 (21.6%)	40 (78.4%)	
Oxaliplatin-based chemotherapy, *n*	135	24 (17.8%)	111 (82.2%)	0.202
Irinotecan-based chemotherapy, *n*	115	28 (24.3%)	87 (75.7%)	
Bevacizumab + FOLFOX, *n*	125	20 (16.0%)	105 (84.0%)	0.088
Bevacizumab + FOLFIRI, *n*	115	28 (24.3%)	87 (75.7%)	
Bevacizumab + CAPOX, *n*	10	4 (40.0%)	6 (60.0%)	
**Treatment response**				
Complete response (CR), *n*	6	1 (16.7%)	5 (83.3%)	0.088
Partial response (PR), *n*	132	19 (14.4%)	113 (85.6%)	
Stable disease (SD), *n*	83	26 (31.3%)	57 (68.7%)	
Progressive disease (PD), *n*	29	6 (20.7%)	23 (79.3%)	

Data are presented as median (interquartile range) for continuous variables and number (percentage) for categorical variables. Continuous variables were compared using the Mann–Whitney U test. Categorical variables were compared using the chi-square test or Fisher’s exact test, as appropriate. All patients received bevacizumab-containing systemic therapy. Chemotherapy backbone was classified as oxaliplatin-based or irinotecan-based according to the regimen administered concomitantly with bevacizumab. Response assessment was performed according to RECIST version 1.1. Progression-free survival (PFS) was defined as the time from initiation of bevacizumab-based therapy to radiological progression or death from any cause. Overall survival (OS) was defined as the time from initiation of bevacizumab-based therapy to death from any cause or last follow-up. Abbreviations: BMI: Body mass index; ECOG: Eastern Cooperative Oncology Group performance status; MSI: Microsatellite instability; MSI-H: Microsatellite instability–high; MSS: Microsatellite stable; RAS: KRAS and/or NRAS mutation; BRAF V600E: BRAF valine-to-glutamic acid substitution at codon 600; PR: Partial response; SD: Stable disease; PD: Progressive disease; PFS: Progression-free survival; OS: Overall survival. †: Molecular data were available only for a subset of patients.

**Table 2:** Progression-Free Survival According to Histology, Peritoneal Metastasis Status, and Chemotherapy Backbone.

Analysis Stratum	Treatment Group	Median PFS (Months)	95% Confidence Interval	Log-Rank χ^2^	*p*-Value
**Overall cohort**	Non-mucinous (*n* = 198)	11.9	10.22–13.58		
	Mucinous (*n* = 52)	22.9	17.95–27.85	23.96	<0.001
**Patients with peritoneal metastasis**	Non-mucinous (*n* = 72)	8.7	6.29–11.12		
	Mucinous (*n* = 35)	23.9	19.81–27.89	18.65	<0.001
**Patients without peritoneal metastasis**	Non-mucinous (*n* = 126)	12.3	11.03–13.57		
	Mucinous (*n* = 17)	14.0	9.51–18.49	1.96	0.162
**Non-mucinous subgroup**	Oxaliplatin-based (*n* = 111)	11.6	8.59–14.67		
	Irinotecan-based (*n* = 87)	12.0	9.59–14.41	0.03	0.863
**Mucinous subgroup**	Oxaliplatin-based (*n* = 24)	16.2	8.18–24.22		
	Irinotecan-based (*n* = 28)	24.1	18.34–29.86	1.72	0.190
**Mucinous histology with peritoneal metastasis**	Oxaliplatin-based (*n* = 13)	23.45	15.61–31.29		
	Irinotecan-based (*n* = 22)	23.85	18.48–29.22	0.01	0.912

Progression-free survival (PFS) was estimated using the Kaplan–Meier method. Log-rank *p*-values for peritoneal metastasis strata represent within-histology comparisons. Events were defined as radiological disease progression or death from any cause. Chemotherapy backbone refers to oxaliplatin-based or irinotecan-based regimens administered in combination with bevacizumab. Subgroups are not mutually exclusive; patients may be represented across different analytical strata according to histology, metastatic pattern, and chemotherapy backbone. Abbreviations: PFS: Progression-free survival.

**Table 3:** Univariate and multivariate Cox proportional hazards analysis for progression-free survival in patients with metastatic colorectal cancer treated with bevacizumab.

Variable	Univariate HR (95% CI)	*p* Value	Multivariate HR (95% CI)	*p* Value
**Age ≥ 65 years**	1.30 (0.97–1.74)	0.085	—	—
**Male sex**	1.01 (0.75–1.36)	0.960	—	—
**BMI ≥ 30 kg/m^2^**	0.78 (0.54–1.13)	0.183	—	—
**ECOG performance status ≥ 2**	1.22 (0.90–1.65)	0.200	—	—
**Mucinous histology**	**0.39 (0.26–0.58)**	**<0.001**	**0.44 (0.25–0.78)**	**0.005**
**Right-sided primary tumor**	1.28 (0.92–1.77)	0.138	—	—
**De novo metastatic disease**	**0.64 (0.47-0.88)**	**0.005**	0.79 (0.52–1.20)	0.264
**Peritoneal metastasis present**	**0.66 (0.48–0.90)**	**0.008**	0.92 (0.60–1.40)	0.693
**Liver metastasis present**	**2.35 (1.63–3.37)**	**<0.001**	1.62 (0.95–2.78)	0.079
**Lung metastasis present**	0.91 (0.68–1.23)	0.547	—	—
**RAS mutation present**	0.99 (0.72–1.36)	0.959	—	—
**BRAF V600E mutation present**	0.64 (0.31–1.32)	0.233	—	—
**MSI-H status**	**0.35 (0.13–0.96)**	**0.040**	0.39 (0.14–1.10)	0.074
**Irinotecan-based chemotherapy (vs oxaliplatin-based)**	0.85 (0.64–1.14)	0.285	—	—
**Bevacizumab used as ≥2nd-line therapy**	1.19 (0.84–1.69)	0.323	—	—

Hazard ratios (HRs) and 95% confidence intervals (CIs) were estimated using Cox proportional hazards regression models. Variables with a *p* value < 0.10 in univariate analysis were entered into the multivariate model. Progression-free survival (PFS) was defined as the time from initiation of bevacizumab-based systemic therapy to radiological disease progression or death from any cause. All covariates were analyzed as binary variables to enhance model stability and avoid overfitting. The proportional hazards assumption was assessed using Schoenfeld residuals. Molecular variables were analyzed using complete-case analysis. Reference categories: Age: <65 years; Sex: Female; BMI: <30 kg/m^2^; ECOG performance status: 0–1; Histology: Non-mucinous; Primary tumor location: Left-sided; Disease presentation: Recurrent metastatic disease; Peritoneal, liver, and lung metastases: Absent; RAS and BRAF: Wild-type; MSI: MSS/MSI-L; HER2: Negative; Chemotherapy backbone: Oxaliplatin-based; Bevacizumab line: First-line therapy. Abbreviations: BMI: Body mass index; ECOG: Eastern Cooperative Oncology Group; MSI-H: Microsatellite instability–high; MSS: Microsatellite stable; RAS: KRAS and/or NRAS mutation; BRAF V600E: BRAF valine-to-glutamic acid substitution at codon 600; HER2: Human epidermal growth factor receptor 2; HR: Hazard ratio; CI: Confidence interval; PFS: Progression-free survival.

**Table 4:** Interaction analysis for progression-free survival in bevacizumab-treated metastatic colorectal cancer.

Interaction Term	HR (95% CI)	*p* for Interaction
**Mucinous histology × Peritoneal metastasis**	**0.45 (0.21–0.97)**	**0.040**
**Mucinous histology × Irinotecan-based chemotherapy**	0.73 (0.38–1.39)	0.341
**Peritoneal metastasis × Irinotecan-based chemotherapy**	1.02 (0.63–1.64)	0.935

Interaction terms were evaluated using Cox proportional hazards regression models including the main effects and corresponding interaction terms. Progression-free survival (PFS) was defined as the time from initiation of bevacizumab-based systemic therapy to radiological disease progression or death from any cause. A statistically significant *p* value for interaction (<0.05) indicates that the effect of chemotherapy backbone or peritoneal metastasis on PFS differs according to histology. Models were adjusted for clinically relevant covariates including age, ECOG performance status, primary tumor location, disease presentation, and molecular characteristics. Abbreviations: HR: Hazard ratio; CI: Confidence interval; PFS: Progression-free survival. These descriptive data were used to support the robustness and interpretability of subgroup and interaction analyses. Abbreviations: PFS: Progression-free survival.

**Table 5:** Univariate and multivariate Cox proportional hazards analysis for overall survival in patients with metastatic colorectal cancer treated with bevacizumab.

Variable	Univariate HR (95% CI)	*p* Value	Multivariate HR (95% CI)	*p* Value
**Age ≥ 65 years**	1.44 (1.05–1.97)	0.022	**1.38 (1.01–1.89)**	**0.041**
**Male sex**	0.93 (0.68–1.27)	0.644	—	—
**BMI ≥ 30 kg/m^2^**	0.86 (0.59–1.26)	0.449	—	—
**ECOG performance status ≥ 2**	1.29 (0.94–1.76)	0.113	—	—
**Mucinous histology**	**0.65 (0.44–0.97)**	**0.036**	0.81 (0.54–1.23)	0.328
**Right-sided primary tumor**	1.05 (0.75–1.47)	0.779	—	—
**De novo metastatic disease**	**0.43 (0.31–0.60)**	**<0.001**	**0.49 (0.35–0.69)**	**<0.001**
**Peritoneal metastasis present**	0.93 (0.68–1.26)	0.624	—	—
**Liver metastasis present**	**2.35 (1.62–3.41)**	**<0.001**	**1.80 (1.21–2.67)**	**0.004**
**Lung metastasis present**	1.34 (0.98–1.84)	0.071	**1.41 (1.01–1.95)**	**0.043**
**RAS mutation present**	1.29 (0.92–1.80)	0.143	—	—
**BRAF V600E mutation present**	0.56 (0.25–1.23)	0.144	—	—
**MSI-H status**	0.57 (0.21–1.58)	0.276	—	—
**Irinotecan-based chemotherapy (vs oxaliplatin-based)**	0.84 (0.62–1.14)	0.266	—	—
**Bevacizumab used as ≥2nd-line therapy**	0.70 (0.48–1.02)	0.066	0.69 (0.47–1.02)	0.062

Hazard ratios (HRs) and 95% confidence intervals (CIs) were estimated using Cox proportional hazards regression models. Variables with a *p* value < 0.10 in univariate analysis were included in the multivariate model. Overall survival (OS) was defined as the time from initiation of bevacizumab-based systemic therapy to death from any cause or last follow-up. All covariates were analyzed as binary variables to improve model stability and avoid overfitting. The proportional hazards assumption was assessed using Schoenfeld residuals. Molecular variables were analyzed using complete-case analysis. Reference categories: Age: <65 years; Sex: Female; BMI: <30 kg/m^2^; ECOG performance status: 0–1; Histology: Non-mucinous; Primary tumor location: Left-sided; Disease presentation: Recurrent metastatic disease; Peritoneal, liver, and lung metastases: Absent; RAS and BRAF: Wild-type; MSI: MSS/MSI-L; HER2: Negative; Chemotherapy backbone: Oxaliplatin-based; Bevacizumab line: First-line therapy. Abbreviations: BMI: Body mass index; ECOG: Eastern Cooperative Oncology Group; MSI: Microsatellite instability; MSI-H: Microsatellite instability–high; MSS: Microsatellite stable; RAS: KRAS and/or NRAS mutation; BRAF V600E: BRAF valine-to-glutamic acid substitution at codon 600; HER2: Human epidermal growth factor receptor 2; HR: Hazard ratio; CI: Confidence interval; OS: Overall survival.

## Data Availability

The data used and analyzed in this study are available from the corresponding author upon reasonable request.
